# Tramadol, captagon and khat use in the Eastern Mediterranean Region: opening Pandora's box

**DOI:** 10.1192/bji.2021.53

**Published:** 2022-08

**Authors:** Hossein Mohaddes Ardabili, Abolfazl Akbari, Parnian Rafei, JL Butner, Riaz Khan, Yasser Khazaal, Abdulmalik Zuhair Arab, Mohammed Rafiq Qazizada, Basma Al-Ansari, Alexander Mario Baldacchino

**Affiliations:** 1 Psychiatry Resident, Psychiatry and Behavioral Sciences Research Center, Mashhad University of Medical Sciences, Mashhad, Iran; and International Society of Addiction Medicine New Professionals Exploration, Training & Education Committee (ISAM NExT), Calgary, Canada; 2 Medical Student, Student Research Committee, Faculty of Medicine, Mashhad University of Medical Sciences, Mashhad, Iran; 3 MSc. Clinical Psychology, Department of Psychology, Faculty of Psychology and Education, University of Tehran, Iran; and International Society of Addiction Medicine New Professionals Exploration, Training & Education Committee (ISAM NExT), Calgary, Canada; 4 Addiction Medicine Specialist, International Society of Addiction Medicine New Professionals Exploration, Training & Education Committee (ISAM NExT), Calgary, Canada; 5 Professor, Psychiatry Specialist, Department of Psychiatry, Frontier Medical College, Affiliated to Bahria University, Abbottabad, Pakistan; 6 Full Professor of Addiction Psychiatry, Addiction Medicine, Department of Psychiatry, Lausanne University Hospital and Lausanne University, Switzerland; 7 PG Researcher, Division of Systems Medicine, School of Medicine, University of Dundee, UK; 8 Dr., Communicable Diseases Control Directorate, National AIDs Control Program, Communicable Diseases Control Directorate, Ministry of Public Health, Kabul, Afghanistan; 9 PhD Addiction Medicine, Addiction Medicine, Sydney Medical School, University of Sydney, NSW, Australia; 10 Medicine, Psychiatry and Addictions Professor, Division of Population and Behavioural Science, School of Medicine, University of St Andrews, UK. Email amb30@st-andrews.ac.uk

**Keywords:** Epidemiology, Eastern Mediterranean region, khat, tramadol, captagon

## Abstract

As defined by the World Health Organization, the Eastern Mediterranean Region (EMR), given its special geopolitical situation and internal/external conflicts, faces an increase in illegal activities such as drug production and trafficking, highlighting the need for a comprehensive understanding of the substance use situation. On the basis of a review of published papers between 2015 and 2021 we briefly review substance use in the EMR with special focus on the emerging drugs pertinent to this region, namely tramadol, captagon and khat.

## Background

According to World Health Organization (WHO) classification, the Eastern Mediterranean Region (EMR) comprises 22 countries with a total population of nearly 679 million.^1^ These countries are: Afghanistan, Bahrain, Djibouti, Egypt, Iran (Islamic Republic of), Iraq, Jordan, Kuwait, Lebanon, Libya, Morocco, Oman, Pakistan, Palestine, Qatar, Saudi Arabia, Somalia, Sudan, Syrian Arab Republic, Tunisia, United Arab Emirates and Yemen.

**Fig. 1:**
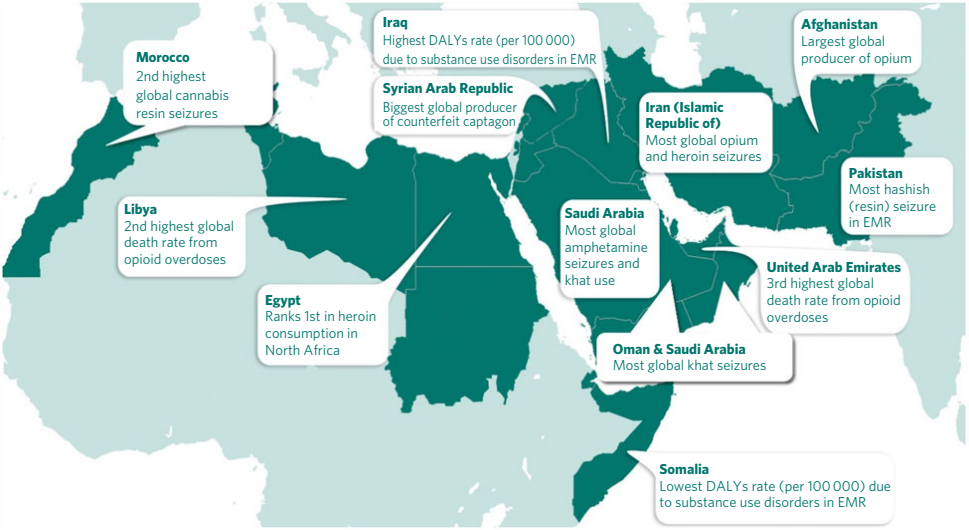
Impact of drugs in the Eastern Mediterranean region (EMR).^4–6^ DALY, disability-adjusted life-year.

**Fig. 2:**
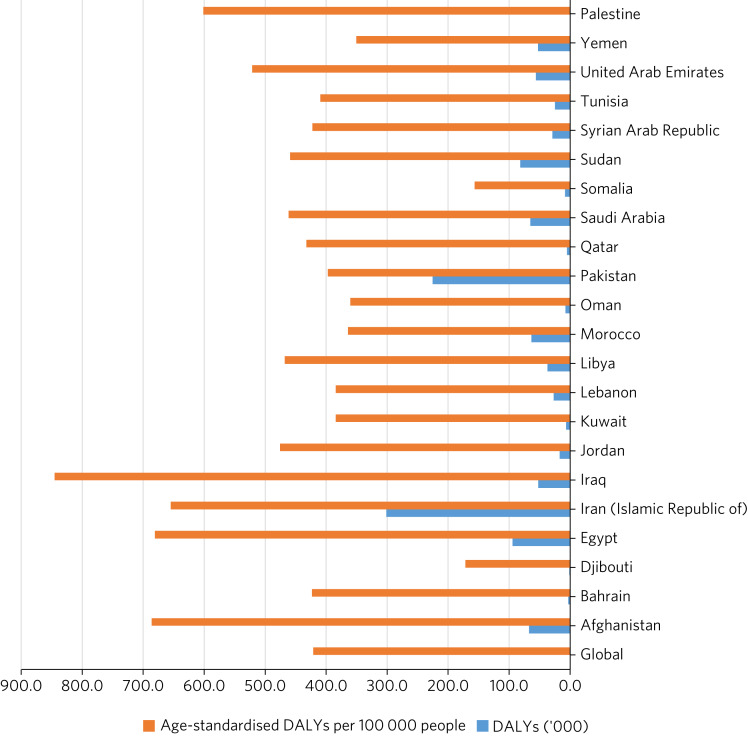
Age-standardised disability-adjusted life-years (DALYs) per 100 000 people (2016) and total DALYs (’000) (2015) in the Eastern Mediterranean region compared with the global estimate.^8,9^ Some data were not available.

The prevailing situation of war, insurgencies, political conflict and civil unrest in many countries of the region has dramatically influenced substance use problems in every aspect, from production and trafficking to availability and pattern of use. This is compounded by the long-standing position of this region as one of the largest opium production sites globally.^2^ These instabilities prevent health and social care systems from providing and sustaining harmonised and integrated effective services. Additionally, increased production and availability of different types of stimulant, especially captagon, has also become a major drug problem in the region^3^ (Fig. 1). From the information available the general situation of substance use in the EMR region indicates that in 2017 about 4.2 million disability-adjusted life-years (DALYs) were lost due to substance use disorders^7^ (Fig. 2). The regional estimated crude death rate due to substance use disorders in 2015 was reported as 1.5 per 100 000 population, which has not changed since 2000.^10^ Although this is lower than the global estimate in 2015 (2.3/100 000), the figures should be carefully considered owing to possible weaknesses in the collection of the epidemiological data.

## Pattern of substance use in the region

The EMR has been the scene of prominent production and seizure of opioids, cocaine, amphetamine-type stimulants and khat.

### Opioids

Opioids remain the main cause of mortality related to use of illicit substances in the region. The second- and third-highest global death rates per 100 000 from opioid overdoses occur in Libya and the United Arab Emirates (UAE) respectively.^11^ Also, opium smoking is a traditional practice in countries such as Afghanistan, Iran and Iraq.

Tramadol is an opioid that is widely used to treat moderate to severe pain and has also been used off-label in the treatment of sexual dysfunction such as premature ejaculation. There is growing evidence of non-medical use of tramadol in the EMR, reflected in the number of people in treatment for tramadol-related problems and the number of tramadol overdose deaths reported in some countries, particularly among young people.^12^ Different studies have concluded that the high levels of misuse of tramadol are a result of its easy availability in pharmacies (including without medication refills in some of these countries) and illicit markets, low price compared with illicit drugs, perceptions among users that tramadol is safe as it is a prescription medication, and the ease with which it can be hidden. Having in mind that less than 10% of tramadol users have a medical source,^13^ the non-medical use of tramadol has been reported by many countries, including Egypt, Iran, Jordan, Lebanon, Libya, Qatar, Saudi Arabia and the UAE. Among Egyptian adolescents, tramadol use is more prevalent than heroin.^14^ Tramadol use in the Iranian general population has been estimated at 4.9% among males and 0.8% among females.^15^ Increasingly, tramadol has been placed under national control in most EMR countries and is therefore only legally accessible by prescription. Such tight control is concerning because it may restrict the medical use of tramadol, particularly in countries of the region where regulatory mechanisms make other opioids (opioid agonist treatments for opiate addiction) less available for medical use.

### Amphetamine-type stimulants, including captagon

The increasing trend of clandestine manufacture and use of amphetamine-type stimulants has become a major concern in countries such as Iran, Morocco and Pakistan. There is also a high demand for fenethylline (captagon) tablets in some countries of the region, especially in Syria, Lebanon and countries in the Arabian Peninsula.^16^ Captagon^®^ was first introduced for its beneficial effects on hyperactivity, depression and narcolepsy, but its addictive and hallucinogenic features made it a popular illegal psychoactive substance.^17^ Captagon^®^ itself is no longer manufactured and counterfeit captagon tablets are mostly combined with amphetamine, caffeine, ephedrine, quinine, theophylline acetaminophen and diphenhydramine and may cause unpredictable complications.^3^ In Saudi Arabia, there are more treatment admissions registered as a result of captagon use than opioid use.^18^

In addition to the established patterns, there have been emerging regional trends of substance use not only related to cultural and geographical expectations in the region but also as a result of the large number of displaced populations. Captagon is an illustration of this trend.^19^ A novel concern about captagon use in Syria and neighbouring countries is that soldiers from all parties to the conflict use the drugs as combat stimulants (‘chemical courage’).^17^ Meantime the current unstable situation in Syria has forced some traffickers to move production of captagon to Libya, Jordan, the UAE, Saudi Arabia and Sudan.^19^

### Khat

*Catha edulis*, commonly known as khat, is a flowering plant growing in the khat belt countries in the Horn of Africa and the Arabian Peninsula. Khat misuse and dependency is still the major illicit substance use-related problem in countries such as Djibouti, Yemen, Somalia and southern provinces of Saudi Arabia.^20^ Most of the effect of chewing khat is thought to come from two chemicals that are structurally related to amphetamine. In Saudi Arabia, khat use is mostly prevalent in the Jazan region in the south of the country on the Yemeni border. A community-based study conducted in the Jazan region reported an overall prevalence of 29% of current khat chewing among respondents. Khat chewing was about four times higher among males than females in both current and lifetime users.^21^ It is reported that nearly 67.9% of adults in Yemen have at least one lifetime episode of khat use.^20^

People who use khat frequently report increased levels of energy, alertness and self-esteem, better ability for communication, sensations of elation, enhanced imaginative ability and a greater capacity for associating ideas. The literature suggests that khat use is associated with personal/public health and social problems. Khat chewing might reduce productivity considering the large amount of time individuals spend on khat chewing. Additionally, another group of studies suggested a possible association between heavy khat consumption and psychosis.^22^

### Multiple substance use

Multiple substance use is a common practice in the EMR and adulterated substances available on the drug market have added to the vulnerability of people with substance use to various health hazards, making the clinical picture of drug intoxication complicated. For example, in some countries of the EMR people have added amphetamine-type stimulant use to opium use – drugs with completely different profiles of intoxication and withdrawal symptoms.^23^ Amphetamine-type stimulant use by patients under methadone maintenance treatment, especially women, is also a potential threat to treatment success.^24^ Lead poisoning among people who use opium has been a problem predominantly observed in Iran due to the impurity of opium products.^25^ The impurity of drugs might be due to the addition of heavy metals by local retailers, to produce heavier packages and therefore higher prices.^26^ The other routine scenario is adding hallucinogens or sedatives or even other pharmacological formulas to improve the experience of the person who uses the drug.^27^ Therefore, many people who have used a single street substance may present with a mixed clinical picture.

## Conclusions

The EMR has a unique nature due to the presence of conflict and emergency in a number of countries. One may hypothesise that a combination of (a) social instability with additional increase in forcibly displaced populations, (b) increased demand in these substances for personal consumption by the affected heterogenous populations in the region and involved combatants, as well as (c) the enhanced production and distribution of these emerging drug markets has synergistically created the 'perfect storm' across the Eastern Mediterranean countries. This heterogeneity is reflected in the hybrid pattern of substance use and the emergence of new trends. The presence of a significant number of populations on the move due to internal displacement or migration affects the capacity of national health systems to effectively manage the evergrowing substance use problem and to meet the needs of the population. Accurate epidemiological data are needed to allow a better understanding of the impact of this problem on both general and marginalised populations.

Key points of this article are summarised in Box 1.

Box 1 Key points

- Within the EMR there is increasing use of opioids (tramadol) and stimulants (captagon and khat) outside the usual cultural and/or medical boundaries for these potentially psychoactive substances.
- Regional political instabilities have fuelled the use, production and trafficking of these substances, potentially creating problems within healthcare systems.
- Being known as the traditional production and trafficking hub for various drugs demands the importance and need for more attention to control the substance use situation in the EMR. This can only be achieved with reliable and accurate data collection.
- The exclusive video abstract (available at: https://vimeo.com/bjpsych/bji-2021-53) also summarizes the key points of this paper.

## Data Availability

Data availability is not applicable to this article as no new data were created or analysed in this study.

## Supplementary material

For supplementary material accompanying this paper visit https://doi.org/10.1192/bji.2021.53. click here to view supplementary material
