# Supplementary material for: Tramadol, captagon and khat use in the Eastern Mediterranean Region: opening Pandora's box
Source: BJPsych Int. 2022 Aug;19(3):58–62. doi: 10.1192/bji.2021.53 (PMC9540563; doi:10.1192/bji.2021.53)
Supplement: Supplementary file 1 [file S2056474021000532sup001.docx]

**Supplementary materials:**

**Street names of drug products in the EMR region**

**Sheesha/Shisheh (Persian: شیشه): –** The Dari term “Sheesha” or the Persian pronunciation “Shisheh”, which directly translates to “glass”, appears to be a common street name for methamphetamine in Afghanistan and Iran. There are other street names for ATS in Iran, but Shisheh is the name most frequently used. It is likely that the term refers to the crystalline appearance of the substance. This should not be confused with the term “shisha” (Arjilah, madā`ah (مداعة), and Hookah) which is used mostly in Arab countries as a street name for waterpipe.

**Tablet K –** “Tablet K” is the street name for a drug with (perceived) stimulant effects sold in Afghanistan. The name seems to be used for a range of tableted products sold on the drug market. It is possible that tablets sold under the street name “tablet K” might contain methamphetamine, MDMA, or a range of other substances.

**“Se Doodi” (Persian;** سه دودی**):** A type of heroin opiate has been introduced to people with severe substance use in northeast Iran Mashhad. According to the clinical features, the euphoric effects of this opiate are reported to be more potent than coal heroin, crystal heroin, and crack heroin.

**“Ashk-e-Huda/Ashk-e-Lily” (Persian: اشک هدی، اشک لیلی):** This term which translates to “tear of Huda/tears of love” came from Pakistan to Afghanistan for a Heroin-based drug. On the back cover of the drug package there was a picture of a lady named Huda, that’s probably why it is called so.

**Street names for amphetamines in Afghanistan:** There are a number of common street names in each region or city; for example, “nakh”, which translates to “yarn”, is used in Kabul for methamphetamine. In Nangarhar province, a street name for methamphetamine is “yakh” which translates to “ice”. In the province of Mazar, drug treatment providers also reported “nabat” (a type of local candy) to be a common street name for methamphetamine, which incidentally is also the brand name of a popular candy sold in Afghanistan.

**Colombian sheesha:** authorities in Afghanistan believe that “Colombian sheesha” is simply another street name for methamphetamine and not connected to Colombia.

**Crystal:** In Afghanistan and Iran, “crystal” is a term commonly used for heroin that is assumed to be of high quality. Iranian Crystal is made in illegal laboratories, with no standardization. Unfortunately, the lack of awareness about the toxicity of this substance has caused health problems among adults and young people who used it freely and fearlessly. Even some Iranian people with substance use believe that the crystal is the crystal-meth (amphetamine based) because of the similarity of the names. In other parts of Iran, the word Crystal widely refers to Iranian Crack which its chemical composition varies not only with methamphetamine content, but also in the adulterants added to cut heroin.

**Crack (Persian: Kerack کراک):** Iranian Crack is a heroin-based narcotic that has been extensively used in Iran. Iranian crack (IC) is completely different from the common crack cocaine. The major ingredients of IC are morphine, caffeine, codeine, thebaine, acetaminophen, and a significant amount of acetyl codeine (1). Another analysis on Iranian crack proved that it contains diacetylmorphine, acetylcodeine, 6-monoacetylmorphine, caffeine, papaverine, noscapine, dextromethorphan, morphine, codeine, phenobarbital, and diazepam (2).

**Kabtagon/Abu Hilalain (Father of the Two Crescent Moons; Arabic ابو هلالین)**: what is sold under this poetic street name of an allusion to the entwining Cs on each pill of Captagon that sometimes contains little but concentrated caffeine and amphetamines. Other common street names in the region include: Abu mlaf, Lajah, Alabyad (white), Al qeshtah (Cream), and Al asfaar (yellow).

**References**

1. Farhoudian A, Sadeghi M, Khoddami Vishteh HR, Moazen B, Fekri M, Rahimi Movaghar A. Component analysis of Iranian crack; a newly abused narcotic substance in iran. Iran J Pharm Res. 2014;13(1):337-44.

2. Akhgari M, Jokar F, Bahmanabadi L, Aleagha AE. Street-level heroin seizures in Iran: a survey of components. Journal of Substance Use. 2012;17(4):348-55.
